# A field evaluation of a new porcine circovirus type 2d and *Mycoplasma hyopneumoniae* bivalent vaccine in herds suffering from subclinical PCV2d infection and enzootic pneumonia

**DOI:** 10.1002/vms3.70001

**Published:** 2024-08-27

**Authors:** Sehyeong Ham, Jeongmin Suh, Chonghan Kim, Byoung‐Joo Seo, Gyeong‐Seo Park, Chanhee Chae

**Affiliations:** ^1^ Department of Veterinary Pathology, College of Veterinary Medicine Seoul National University Seoul Republic of Korea; ^2^ WOOGENE B&G CO., LTD. Seoul Republic of Korea

**Keywords:** enzootic pneumonia, *Mycoplasma hyopneumoniae*, porcine circovirus type 2d, porcine circovirus‐associated diseases, vaccine

## Abstract

**Background:**

This field efficacy study was designed to determine the efficacy of a new bivalent vaccine containing porcine circovirus type 2d (PCV2d) and *Mycoplasma hyopneumoniae* at three independent pig farms.

**Methods:**

Three pig farms were selected based on their history of subclinical PCV2 infection and enzootic pneumonia. Each farm housed a total of 40, 18‐day‐old pigs that were randomly allocated to 1 of 2 treatment groups. Pigs were administered a 2.0 mL dose of the bivalent vaccine intramuscularly at 21 days of age in accordance with the manufacturer's recommendations, whereas unvaccinated pigs were administered a single dose of phosphate‐buffered saline at the same age.

**Results:**

Clinically, the average daily weight gain of vaccinated groups was significantly higher (*p* < 0.05) than those of unvaccinated animals during the growing (70–112 days of age), finishing (112–175 days of age) and overall (3–175 days of age) stages of production. Vaccinated animals elicited neutralizing anti‐PCV2 antibodies and PCV2d‐specific interferon‐γ secreting cells (IFN‐γ‐SC), which reduced the amount of PCV2d genomic copies in blood and reduced lymphoid lesions severity when compared with unvaccinated animals. Similarly, vaccinated animals elicited *M. hyopneumoniae*‐specific IFN‐γ‐SC, which reduced the amount of *M. hyopneumoniae* in the larynx and reduced lung lesions severity.

**Conclusions:**

The result of the field trial demonstrated that the bivalent vaccine was efficacious in the protection of swine herds suffering from subclinical PCV2d infection and enzootic pneumonia.

## Graphical abstract

This study investigates the efficacy of a new bivalent vaccine targeting porcine circovirus type 2d (PCV2d) and *Mycoplasma hyopneumoniae* across three pig farms with histories of subclinical PCV2 infections and enzootic pneumonia. Vaccinated pigs showed higher average daily weight gains, an increased production of neutralizing anti‐PCV2 antibodies and a reduction in the severity of lymphoid and lung lesions compared to unvaccinated pigs.

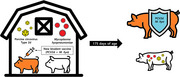

## 1 INTRODUCTION

Porcine circovirus type 2 (PCV2) and *Mycoplasma hyopneumoniae* are some of the most economically important swine disease pathogens (Chae, 2016; Segalés, Allan et al., 2005). PCV2 is the principal etiologic agent of porcine circovirus‐associated disease (PCVAD), which was originally described as post‐weaning multisystemic wasting syndrome (Segalés, Allan et al., 2005). Since the introduction of PCV2 vaccines in 2007, the clinical forms of PCVAD have become less common in the field, whereas subclinical PCV2 infection has become the prevalent form of PCV2 disease (Segalés, 2012). Subclinical infection of pigs with PCV2 resulted in growth performance reduction and an increased susceptibility to other co‐infections (Alarcon et al., 2013). *M. hyopneumoniae* is the primary etiologic agent of enzootic pneumonia, a chronic respiratory disease characterized by a dry cough, growth retardation and reduced feed efficiency. Enzootic pneumonia causes considerable economic loss in all areas where pigs are raised (Maes et al., 1996).

The majority of current commercially available PCV2 vaccines were developed between 1999 and 2005. During this period, PCV2a was the predominant PCV2 field strain, and little was known about additional PCV2 genotypes and their importance. Therefore, the majority of commercially available PCV2 vaccines are based on PCV2a and/or the PCV2b that followed in emergence (Chae, 2012). In 2010, a novel mutation was first reported in China and designated as mutant PCV2b and was later identified in the United States and Korea in 2014 (Guo et al., 2012; Seo et al., 2014; Xiao et al., 2012). This mutant was later renamed as PCV2d. The main evolutionary changes in the genotypes of PCV2 field strains over time occurred between the predominant PCV2a to PCV2b and again from PCV2b to PCV2d (Franzo & Segales, 2018).

PCV2d has replaced PCV2a and PCV2b as the predominant genotype in essentially all global pig populations throughout Asia, North and South America, and Europe (Franzo et al., 2016; Xiao et al., 2015). Taking current field conditions into account, bivalent vaccination with PCV2d and *M. hyopneumoniae* is a valuable strategic tool for controlling porcine respiratory disease complex by these two pathogens. A novel bivalent vaccine containing PCV2d and *M. hyopneumoniae* antigen was recently introduced into the swine commercial market (Ham et al., 2023). The objective of this study was to evaluate the efficacy of this new bivalent vaccine, with a focus on growth performance in swine herds suffering from subclinical PCV2d infection and enzootic pneumonia.

## 2 MATERIALS AND METHODS

### 2.1 Farm history

The clinical field trial was conducted on three farms. Farms were labelled ‘A, B and C’ and were 350‐, 360‐ and 400‐sow (respectively) farrow‐to‐finish swine operations with an all‐in‐all‐out production system. Sows from these three farms were not immunized for either PCV2 or *M. hyopneumoniae*. The porcine reproductive and respiratory syndrome virus (PRRSV) status was stable as active PRRSV was undetected in circulation. Sows and piglets were not immunized against PRRSV, whereas all piglets received vaccinations for PCV2 and *M. hyopneumoniae* at 3 weeks of age.

Each farm consistently experienced respiratory distress as indicated by poor growth rate in the late post‐weaning and growing stages of production. Clinical signs first appeared at approximately 8–11 weeks of age and reached peak mortality (farm A = approximately 3%–5%, farm B = 3%–4% and farm C = 3%–6%) between 10 and 15 weeks of age.

Three farms were selected based on their active status of containing subclinical PCV2 infection and enzootic pneumonia. PCV2d was detected in serum from three pigs within each of these three farms, where log_10_ DNA copies/mL ranged from 2.44 to 3.42. These values were large enough to diagnose the pigs with subclinical PCV2 infection (Segalés, Allan et al., 2005). *M. hyopneumoniae* serology was positive in serum samples from pigs aged between 7 and 15 weeks. A lung examination was performed at the slaughterhouse, which confirmed that 40% of these 30 seropositive pigs had mycoplasmal pneumonia lesions.

### 2.2 Field trial design

The results of this field study are intended for registration and therefore all procedures strictly adhered to the guidelines of the Republic of Korea's Animal, Plant and Fisheries Quarantine and Inspection Agency (QIA, http://www.qia.go.kr). QIA protocols mandate that a total of 20 pigs were assigned to each study group. Study design considerations included randomization, personnel blinding, and that the animals were both weight‐matched and sex‐matched under a controlled clinical field trial format. To minimize sow variation, eight 18‐day‐old pigs were randomly selected from five total sows. A total of 120 pigs were used for the entire study. Forty pigs per farm were randomly divided into 2 groups within each farm (20 pigs per group; 10 = male and 10 = female) using the random number generator function (Excel, Microsoft Corporation).

At 0 days post‐vaccination (dpv, 21 days of age), pigs in the VacA, VacB and VacC groups received a 2.0 mL dose of bivalent vaccine containing PCV2d and *M. hyopneumoniae* (IMMUNIS DMVac, WOOGENE B&G CO. LTD.) by an intramuscular route in the neck muscle (Ham et al., 2023). IMMUNIS DMVac (WOOGENE B&G CO. LTD.) is composed of the inactivated whole PCV2d strain SNUVR201901 and *M. hyopneumoniae* strain WGB‐Mhp bacterin (Ham et al., 2023).

Each farm received a different serial of the vaccine as follows: farm A = serial no: F001, expiration date: 10 May 2024, farm B = serial no: F002, expiration date: 10 May 2024 and farm C = serial no: F003, expiration date: 10 May 2024. Pigs in the UnVacA, UnVacB and UnVacC groups received a 2.0 mL dose of phosphate‐buffered saline (0.01 M, pH 7.4) as described above.

At 7 dpv (28 days of age), pigs from the vaccinated and unvaccinated groups were commingled and randomly assigned into 4 pens (10 pigs per pen). All pens were identical in design with equipment including free access to water and feed. Five pigs from each group were randomly selected and euthanized for necropsy at 112 days of age. The rest of pigs from each group were euthanized for necropsy at 175 days of age. Pigs were sedated by an intravenous injection of sodium pentobarbital and then euthanized by electrocution as previously described (Beaver et al., 2001). Lung, liver, tonsil, kidney, spleen, small and large intestine and superficial inguinal lymph node tissues were collected from each pig at the time of necropsy. Tissues were fixed for 24 h in 10% neutral buffered formalin, routinely processed and embedded in paraffin.

### 2.3 Sample collection

Blood and laryngeal swabs were collected at 0 (21 days old), 28 (49 days old), 49 (70 days old), 91 (112 days old) and 154 (175 days old) dpv.

### 2.4 Clinical observations

The pigs were monitored daily for abnormal clinical signs and scored weekly using scores ranging from 0 (normal) to 6 (severe dyspnea and abdominal breathing) (Halbur et al., 1995). Observers were blinded to vaccination and type of vaccine status. Mortality rate was calculated as the number of pigs that died divided by the number of pigs initially assigned to that group within batch. Pigs that died or were culled throughout the study were necropsied. Evaluation of injection site reaction, including palpation, was performed 24 h post‐vaccination.

### 2.5 Average daily weight gain

The live weight of each pig was measured at 21 (0 dpv), 70 (49 dpv), 112 (91 dpv) and 175 (154 dpv) days of age. The average daily weight gain (ADWG; grams/pig/day) was analysed over three time periods: (i) between 21 and 70 days old, (ii) between 70 and 112 days old, (iii) between 112 and 175 days old and (iv) between 21 and 175 days old. ADWG during the different production stages was calculated as the difference between the starting and final weight divided by the duration of the stage. Data for dead or removed pigs were included in the calculation.

### 2.6 Quantification of PCV2d DNA

DNA will be extracted from serum, tissue, nasal and faecal samples using the commercial kit (QIAamp DNA Mini Kit, QIAGEN) to quantify PCV2d genomic DNA copy numbers by real‐time PCR (Jeong et al., 2015).

### 2.7 Quantification of *M. hyopneumoniae* DNA in larynx

DNA was extracted from laryngeal swabs using the commercial kit (QIAamp DNA Mini Kit, QIAGEN) to quantify the *M. hyopneumoniae* genomic DNA copy numbers by real‐time PCR (Dubosson et al., 2004).

### 2.8 Serology

The serum samples were tested using the commercially available PCV2 (INgezim CIRCO IgG, Ingenasa) and *M. hyopneumoniae* (M. hyo. Ab test, IDEXX Laboratories Inc.). Samples were considered positive for PCV2 antibodies if the sample‐to‐positive (S/P) ratio was ≥0.3 and for *M. hyopneumoniae* antibody if S/P ratio was ≥0.4 in accordance with the manufacturer's instructions for each kit. The serum samples were tested using serum virus neutralization test against PCV2d (Fort et al., 2009; Pogranichnyy et al., 2000; Shen et al., 2010).

### 2.9 Enzyme‐linked immunospot assay

Enzyme‐linked immunospot (ELISPOT) assay was conducted to measure the numbers of PCV2d‐specific and *M. hyopneumoniae*‐specific interferon‐γ secreting cells (IFN‐γ‐SC) (Jeong et al., 2015, 2018). The numbers of PCV2d‐ and *M. hyopneumoniae*‐specific IFN‐γ‐SC were determined in peripheral blood mononuclear cells (PBMCs). The IFN‐γ positive spots on the membranes were imaged, analysed and counted using an automated ELISPOT Reader (AID ELISPOT Reader, AID GmbH). The results were expressed as the numbers of IFN‐γ‐SC per million PBMC. ELISPOT assay was done in duplicate.

### 2.10 Pathology

The severity of macroscopic lung lesions was scored to estimate the percentage of the lung affected by pneumonia. The scoring was done by two pathologists (Chae and one graduate student) at the Seoul National University (Seoul, Republic of Korea). For the entire lung, 100 points were be assigned as follows: 10 points each to the right cranial lobe, right middle lobe, left cranial lobe and left middle lobe, 27.5 points each to the right caudal lobe and left caudal lobe and 5 points to the accessory lobe (Halbur et al., 1995). Two veterinary pathologists then examined the collected lung and lymphoid tissue sections and scored the severity of peribronchiolar and perivascular lymphoid tissue hyperplasia by mycoplasmal pneumonia lesions (0–6) (Opriessnig et al., 2004). Lymphoid lesion severity was scored (0–5) based on lymphoid depletion and granulomatous inflammation (Kim & Chae, 2004).

### 2.11 Statistical analysis

Real‐time PCR and neutralizing antibody data were recalculated into log_10_ and log_2_ values, respectively, prior to statistical analysis. The collected data were assessed by the Shapiro–Wilk test for a normal distribution. Then, Student's *t*‐test was performed to determine if there were statistically significant differences among different groups at each time point. In case of the normality assumption was not met, Mann–Whitney test was performed to compare the differences among different groups. The value of *p* < 0.05 was considered significant and reported in *p*‐values.

## 3 RESULTS

### 3.1 Clinical signs

**FIGURE 1:**
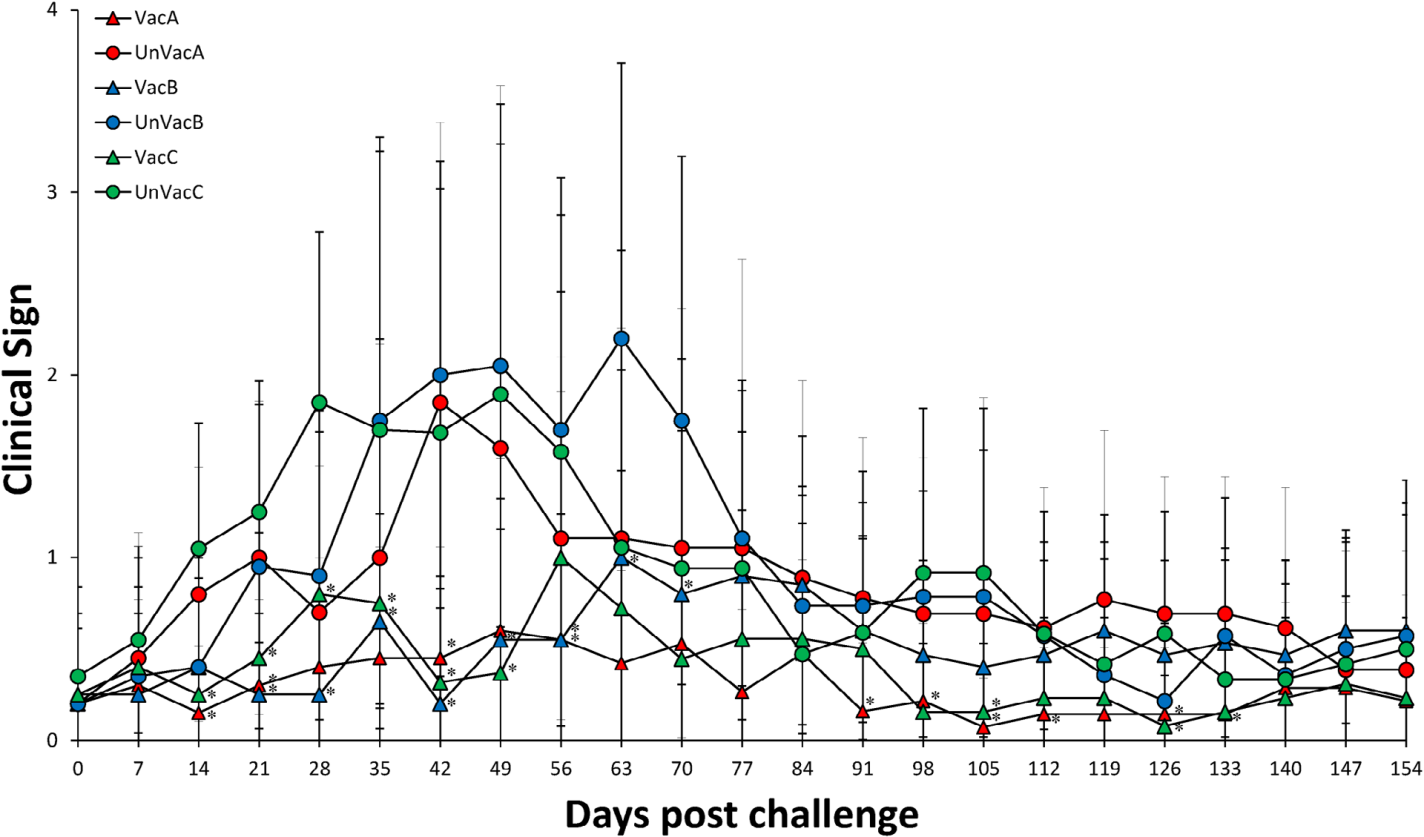
Mean respiratory score from vaccinated and unvaccinated groups in farms A, B and C. Variation is expressed as the standard deviation. ^*^Significant difference (*p* < 0.05) between vaccinated and unvaccinated group within the same farm.

Vaccinated animals within farm A were significantly (*p* < 0.05) lower in respiratory clinical signs compared to unvaccinated animals at 14, 21, 42, 56, 91, 105, 112, 126 and 133 dpv. Respiratory clinical signs of farm B vaccinated animals were significantly (*p *< 0.05) lower compared to those of unvaccinated animals from 21 to 70 dpv. Respiratory clinical signs of farm C vaccinated animals were significantly (*p* < 0.05) lower compared to those of unvaccinated animals from 14 to 49 and at 98, 105 and 126 dpv (Figure 1).

### 3.2 Average daily weight gain

A difference in mean body weight was not observed between vaccinated and unvaccinated animals at the time of vaccination on all three farms. In farms A and B, vaccinated animals were significantly (*p *< 0.05) heavier in body weight than the unvaccinated animals at 112 (91 dpv) and 175 (154 dpv) days of age. Vaccinated animals measured significantly (*p *< 0.05) higher in ADWG than unvaccinated animals between 70 and 112, between 112 and 175 and between 21 and 175 days of age. Vaccinated animals in farm C were significantly (*p *< 0.05) heavier in body weight than unvaccinated animals at 70 (49 dpv), 112 (91 dpv) and 175 (154 dpv) days of age. Vaccinated animals were significantly (*p *< 0.05) higher in ADWG than unvaccinated animals between 70 and 112 and between 21 and 175 days of age (Table 1).

**TABLE 1:** Average daily weight gain (ADWG), body weight, mortality rate and pathology between vaccinated (Vac) and unvaccinated (UnVac) animals on three farms.

		Farm A		Farm B		Farm C	
	Age (day)	VacA	UnVacA	VacB	UnVacB	VacC	UnVacC
ADWG (gram/pig/day)							
	21–70	391 ± 49	372 ± 37	375 ± 46	348 ± 56	363 ± 35	343 ± 23
	70–112	792 ± 74^a^	688 ± 63	789 ± 73^a^	668 ± 68	742 ± 26^a^	663 ± 41
	112–175	839 ± 71^a^	776 ± 57	835 ± 67^a^	776 ± 40	843 ± 27	827 ± 51
	21–175	679 ± 26^a^	622 ± 17	677 ± 32^a^	611 ± 12	658 ± 15^a^	628 ± 21
Body weight (kg)							
	21	5.9 ± 0.5	5.9 ± 0.4	6.1 ± 0.3	6.0 ± 0.6	6.0 ± 0.7	6.0 ± 0.6
	70	25.0 ± 2.5	24.1 ± 1.7	24.4 ± 2.2	23.2 ± 2.6	23.8 ± 1.7^a^	22.7 ± 1.0
	112	58.1 ± 3.2^a^	52.9 ± 2.4	57.6 ± 2.1^a^	51.2 ± 1.4	55.0 ± 2.1^a^	50.6 ± 1.9
	175	110.9 ± 4.3^a^	101.6 ± 2.6	110.2 ± 5.1^a^	100.2 ± 2.0	107.6 ± 2.0^a^	102.6 ± 3.1
Mortality rate		1/20	2/20	0/20	1/20	2/20	3/20
Lung lesion score							
Macroscopic	112	14 ± 8.22^a^	38 ± 8.37	14 ± 11.40^a^	47 ± 12.54	23 ± 16.05^a^	58 ± 14.40
	175	19 ± 14.54^a^	49 ± 14.97	20 ± 22.61^a^	65 ± 16.67	25 ± 26.71^a^	58 ± 25.22
Microscopic	112	1.2 ± 0.79^a^	3.0 ± 0.59	1.4 ± 0.89^a^	3.7 ± 0.48	1.2 ± 0.79^a^	4.0 ± 0.75
	175	1.3 ± 0.79^a^	3.4 ± 0.71	1.6 ± 0.98^a^	3.8 ± 0.86	1.1 ± 0.95^a^	2.1 ± 0.72
Lymphoid lesion	112	1.2 ± 0.67^a^	2.4 ± 0.20	0.8 ± 0.32^a^	2.6 ± 0.26	1.7 ± 0.30^a^	2.8 ± 0.78
score	175	0.5 ± 0.54^a^	2.2 ± 0.72	0.9 ± 0.69^a^	2.8 ± 0.86	0.7 ± 0.60^a^	2.1 ± 0.65
PCV2‐antigen	112	1.4 ± 1.79^a^	9.5 ± 5.93	3.13 ± 3.86^a^	9.07 ± 0.43	2.0 ± 1.39^a^	9.1 ± 6.31
positive cells	175	0.6 ± 0.83^a^	4.5 ± 2.47	0.4 ± 0.89^a^	3.7 ± 2.54	0.6 ± 1.34^a^	3.7 ± 2.32
PCV2 genomic copy number by tissue (Log_10_ copies/mL)							
Lung	112	0.7 ± 1.5^a^	3.6 ± 2.1	0.7 ± 1.4^a^	3.9 ± 2.2	1.6 ± 2.2	3.6 ± 2.2
	175	0.2 ± 0.8	1.3 ± 2.0	0.7 ± 1.5^a^	1.9 ± 2.3	0.9 ± 1.8	1.2 ± 2.2
Tonsil	112	0.8 ± 1.7^a^	4.4 ± 2.6	2.4 ± 2.2^a^	6.0 ± 1.0	1.7 ± 2.4^a^	4.9 ± 1.2
	175	0.6 ± 1.6	2.2 ± 2.9	0.6 ± 1.6	2.8 ± 2.9	1.5 ± 2.3	1.9 ± 2.4
Lymph node	112	0.7 ± 1.6^a^	4.9 ± 2.9	2.2 ± 2.0^a^	5.8 ± 0.8	1.8 ± 2.4^a^	5.1 ± 0.6
	175	0.6 ± 1.6^a^	2.5 ± 2.9	1.0 ± 2.1	2.9 ± 3.1	1.6 ± 2.5	2.0 ± 3.0

^a^ Significant difference (*p* < 0.05) between vaccinated and unvaccinated groups within the same farm.

### 3.3 Mortality

Mortality at all farms was primarily related to infection with PCV2d and *M. hyopneumoniae* in unvaccinated animals. One 77‐day‐old vaccinated pig from farm A died of unknown acute haemorrhagic enteritis, one 68‐day‐old unvaccinated pig died of enzootic pneumonia caused by a co‐infection with *M. hyopneumoniae* and *Pasteurella multocida*, and one unvaccinated 95‐day‐old pig died of enzootic pneumonia caused by a co‐infection with *M. hyopneumoniae* and *Trueperella pyogenes*. One 88‐day‐old unvaccinated pig from farm B died of enzootic pneumonia caused by *M. hyopneumoniae* and *T. pyogenes*. Two vaccinated pigs died during the study in farm C; one at 55‐day‐old from streptococcal meningitis, and one 76‐day‐old from Glasser's disease caused by *Glaesserella parasuis* infection. Three unvaccinated pigs also died in farm C during the study; one 54‐day‐old from pneumonia caused by a co‐infection with PCV2d and *Staphylococcus aureus*, and two from pneumonia caused by co‐infection with PCV2d, *M. hyopneumoniae* and *P. multocida* at 77 and 82 days of age.

### 3.4 Quantification of PCV2 DNA

**FIGURE 2:**
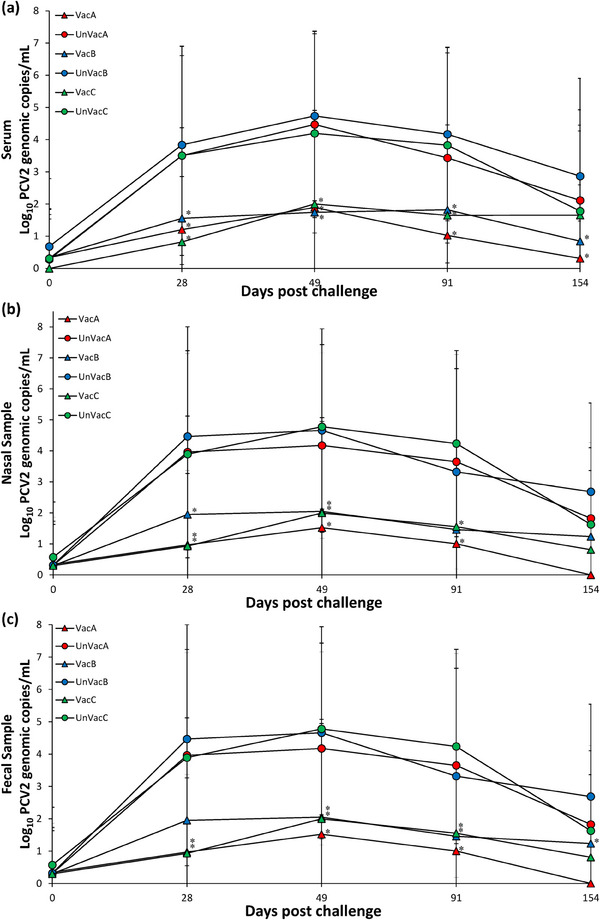
Mean values of the genomic copy number of porcine circovirus type 2d (PCV2d) DNA in serum (a), nasal (b) and faecal (c) samples of pigs from vaccinated and unvaccinated groups in farms A, B and C. Variation is expressed as the standard deviation. ^*^Significant difference (*p* < 0.05) between vaccinated and unvaccinated group within the same farm.

In farm A, vaccinated animals had a significantly (*p* < 0.05) lower amount of PCV2d genomic copies in their blood compared to those of unvaccinated animals at 28, 49 and 154 dpv. In farm B, vaccinated animals had significantly (*p* < 0.05) lower amount of PCV2d genomic copies in their blood compared to those of unvaccinated animals at 28, 49, 91 and 154 dpv. In farm C, vaccinated animals had a significantly (*p* < 0.05) lower amount of PCV2d genomic copies in their blood compared to those of unvaccinated animals at 28 and 49 dpv (Figure 2a).

In farms A and C, vaccinated animals had a significantly (*p* < 0.05) lower amount of PCV2d genomic copies in nasal samples compared to those of unvaccinated animals at 28, 49 and 91 dpv. In farm B, vaccinated animals had a significantly (*p* < 0.05) lower amount of PCV2d genomic copies in their blood compared to those of unvaccinated animals at 28 and 49 dpv (Figure 2b).

In farms A and C, vaccinated animals had a significantly (*p* < 0.05) lower amount of PCV2d genomic copies in oral samples compared to those of unvaccinated animals at 28, 49 and 91 dpv. In farm B, vaccinated animals had a significantly (*p* < 0.05) lower amount of PCV2d genomic copies in their blood compared to those of unvaccinated animals at 28, 49, 91 and 154 dpv (Figure 2c).

In farms A and B, vaccinated animals had a significantly (*p* < 0.05) lower amount of PCV2d genomic copies in lung tissues compared to those of unvaccinated animals at 91 dpv. In farm A, vaccinated animals had a significantly (*p* < 0.05) lower amount of PCV2d genomic copies in lung tissues compared to those of unvaccinated animals at 154 dpv (Table 1).

In farms A, B and C, vaccinated animals had a significantly (*p* < 0.05) lower amount of PCV2d genomic copies in tonsillar and lymph node tissues compared to those of unvaccinated animals at 91 dpv. In farm A, vaccinated animals had a significantly (*p* < 0.05) lower amount of PCV2d genomic copies in lymph node tissues compared to those of unvaccinated animals at 154 dpv (Table 1).

### 3.5 Quantification of *M. hyopneumoniae* DNA

**FIGURE 3:**
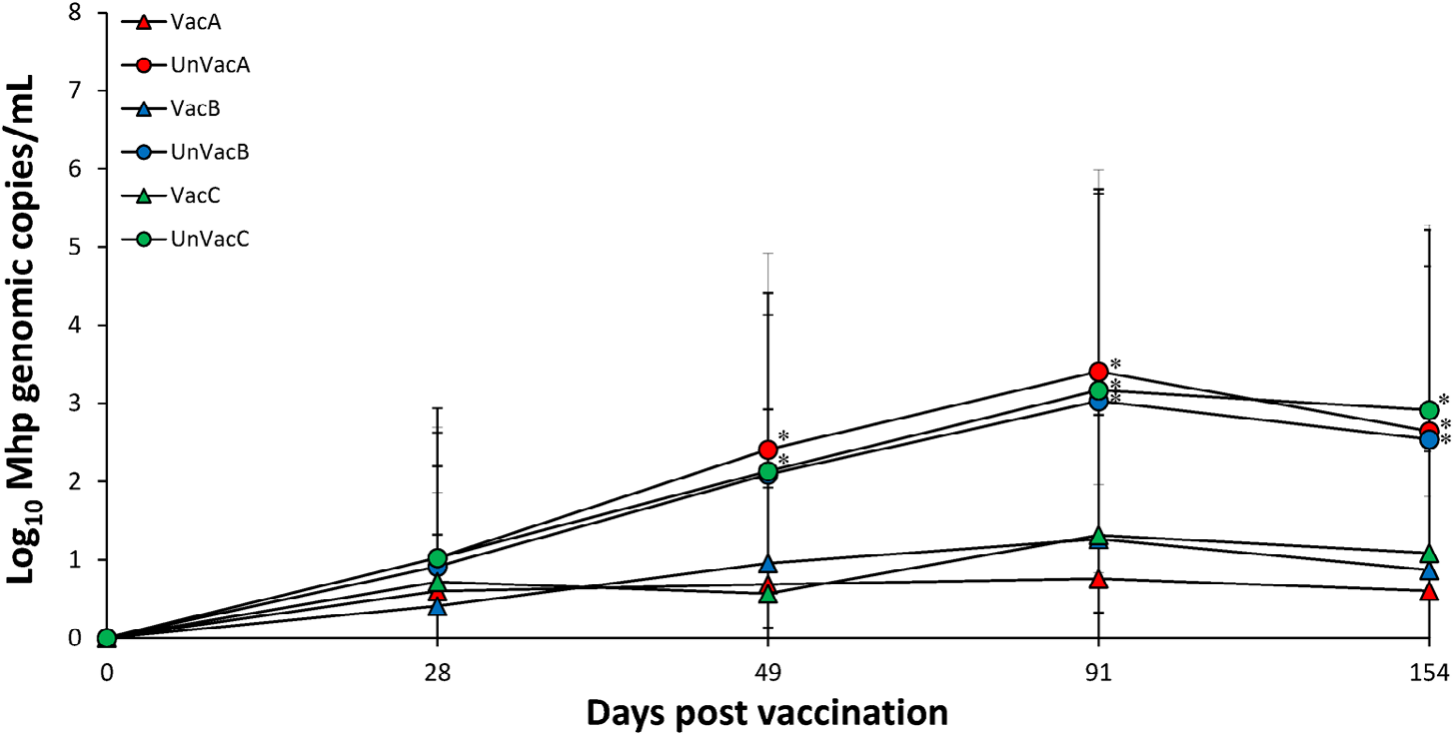
Mean values of the genomic copy number of *Mycoplasma hyopneumoniae* DNA in larynx of pigs from vaccinated and unvaccinated groups in farms A, B and C. Variation is expressed as the standard deviation. ^*^Significant difference (*p* < 0.05) between vaccinated and unvaccinated group within the same farm.

Vaccinated animals from farms A and C had significantly (*p* < 0.05) lower amounts of laryngeal *M. hyopneumoniae* genomic copies compared to those of unvaccinated animals at 49, 91 and 154 dpv. Vaccinated animals from farm B had significantly (*p *< 0.05) lower amounts of laryngeal *M. hyopneumoniae* genomic copies compared to those of unvaccinated animals at 91 and 154 dpv (Figure 3).

### 3.6 Immune responses against PCV2

**FIGURE 4:**
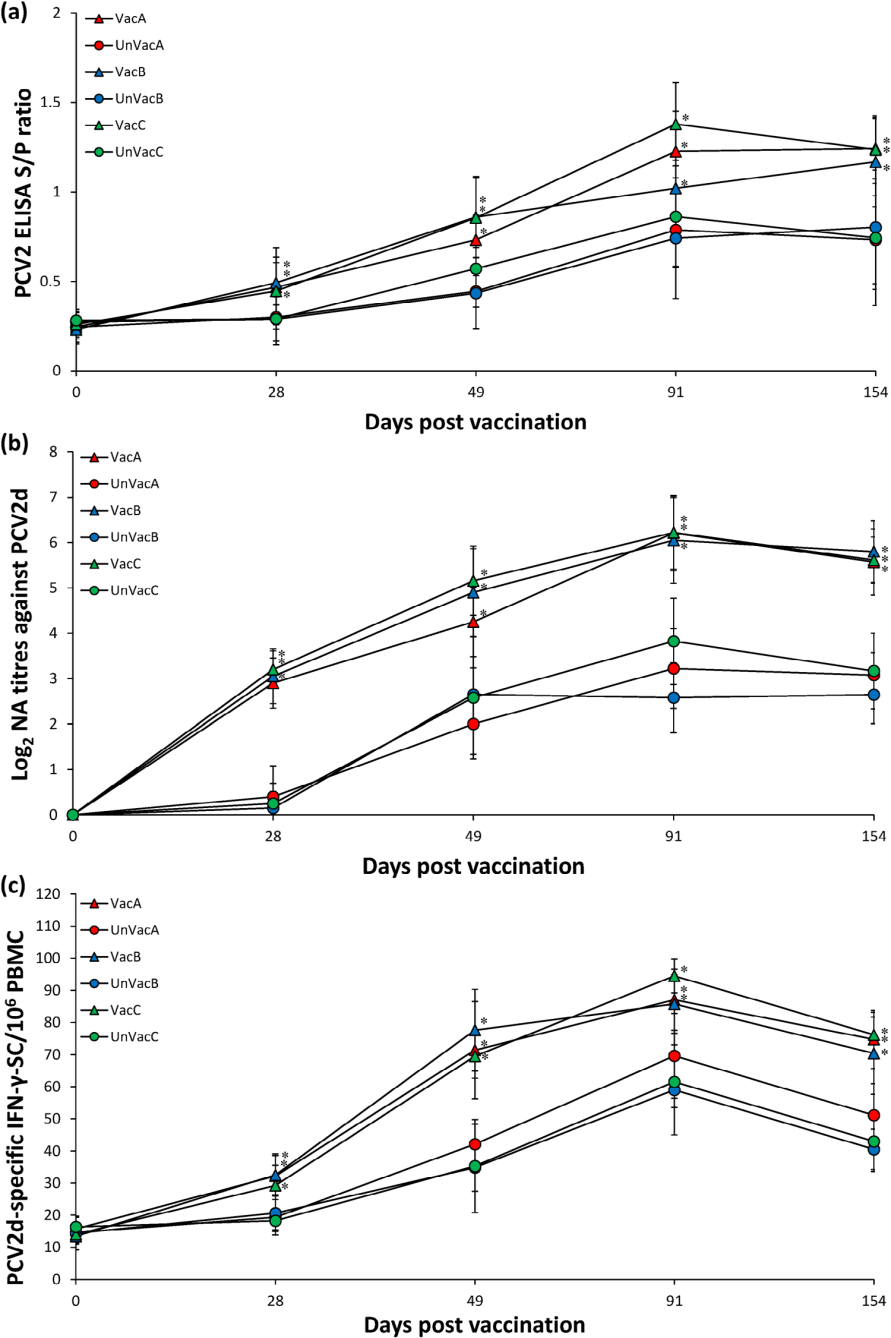
Mean values of the porcine circovirus type 2 (PCV2) ELISA titres (a), the PCV2d‐specific neutralizing antibodies (b) and PCV2d‐specific IFN‐γ‐SC/10^6^ peripheral blood mononuclear cell (PBMC) (c) from vaccinated and unvaccinated groups in farms A, B and C. Variation is expressed as the standard deviation. ^*^Significant difference (*p* < 0.05) between vaccinated and unvaccinated group within the same farm.

Immune responses against PCV2 were consistent in animals across the evaluated three farms during this field trial. Vaccination of animals significantly (*p* < 0.05) increased the levels of PCV2 antibody (Figure 4a), neutralizing PCV2d antibody (Figure 4b) and PCV2d‐specific IFN‐γ‐SC (Figure 4c) compared to unvaccinated animals at 28, 49, 91 and 154 dpv.

### 3.7 Immune responses against *M. hyopneumoniae*

**FIGURE 5:**
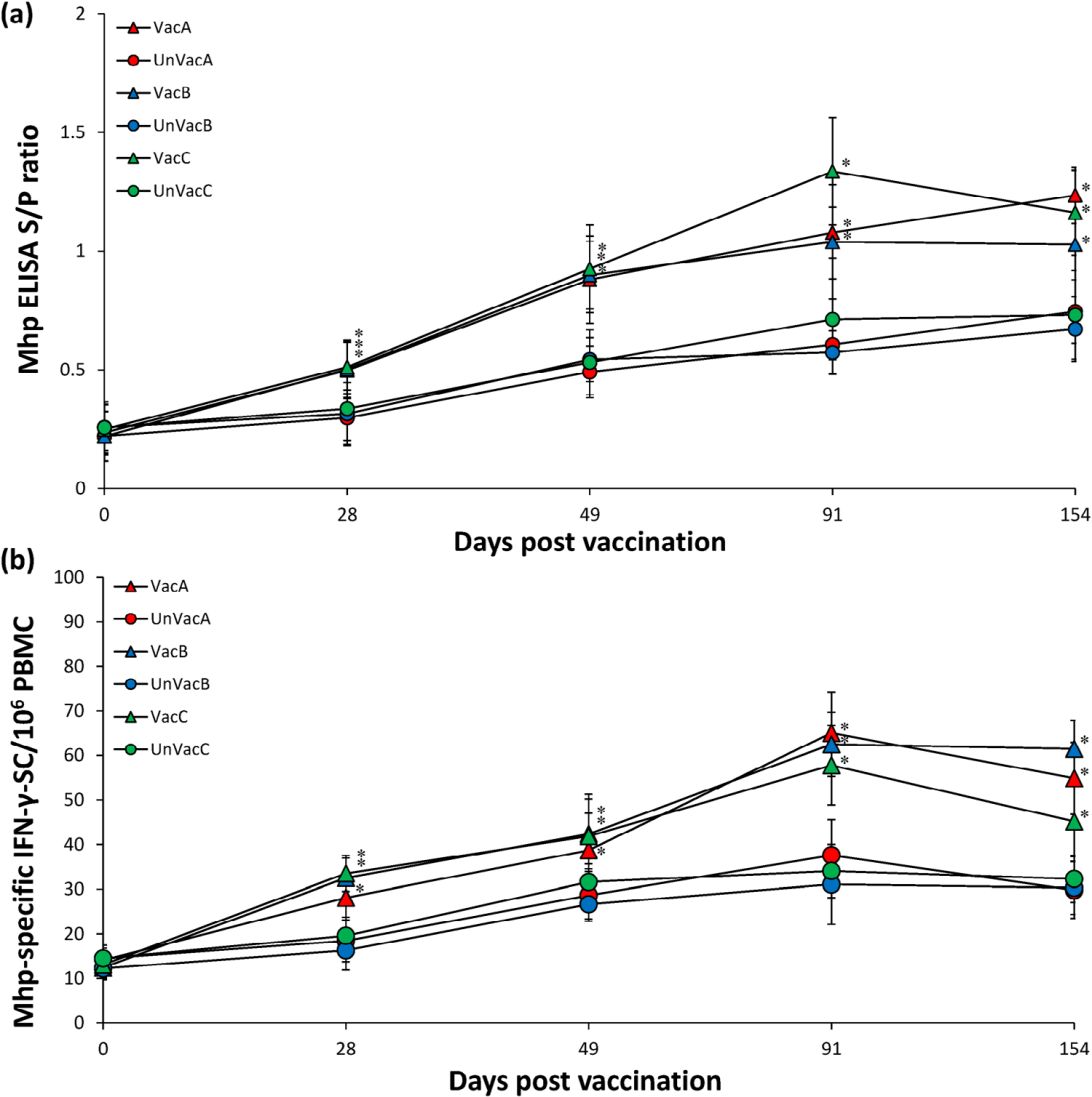
Mean values of the *Mycoplasma hyopneumoniae* ELISA sample‐to‐positive (S/P) ratio (a) and *M. hyopneumoniae*‐specific IFN‐γ‐SC/10^6^ peripheral blood mononuclear cell (PBMC) (b) from vaccinated and unvaccinated groups in farms A, B and C. Variation is expressed as the standard deviation. ^*^Significant difference (*p* < 0.05) between vaccinated and unvaccinated group within the same farm.

Immune responses against *M. hyopneumoniae* were consistent in animals across the evaluated three farms during this field trial. Vaccination of animals significantly (*p* < 0.05) increased the levels of *M. hyopneumoniae* antibody (Figure 5a) and *M. hyopneumoniae*‐specific IFN‐γ‐SC (Figure 5b) compared to unvaccinated animals at 28, 49, 91 and 154 dpv.

### 3.8 Pathology

**FIGURE 6:**
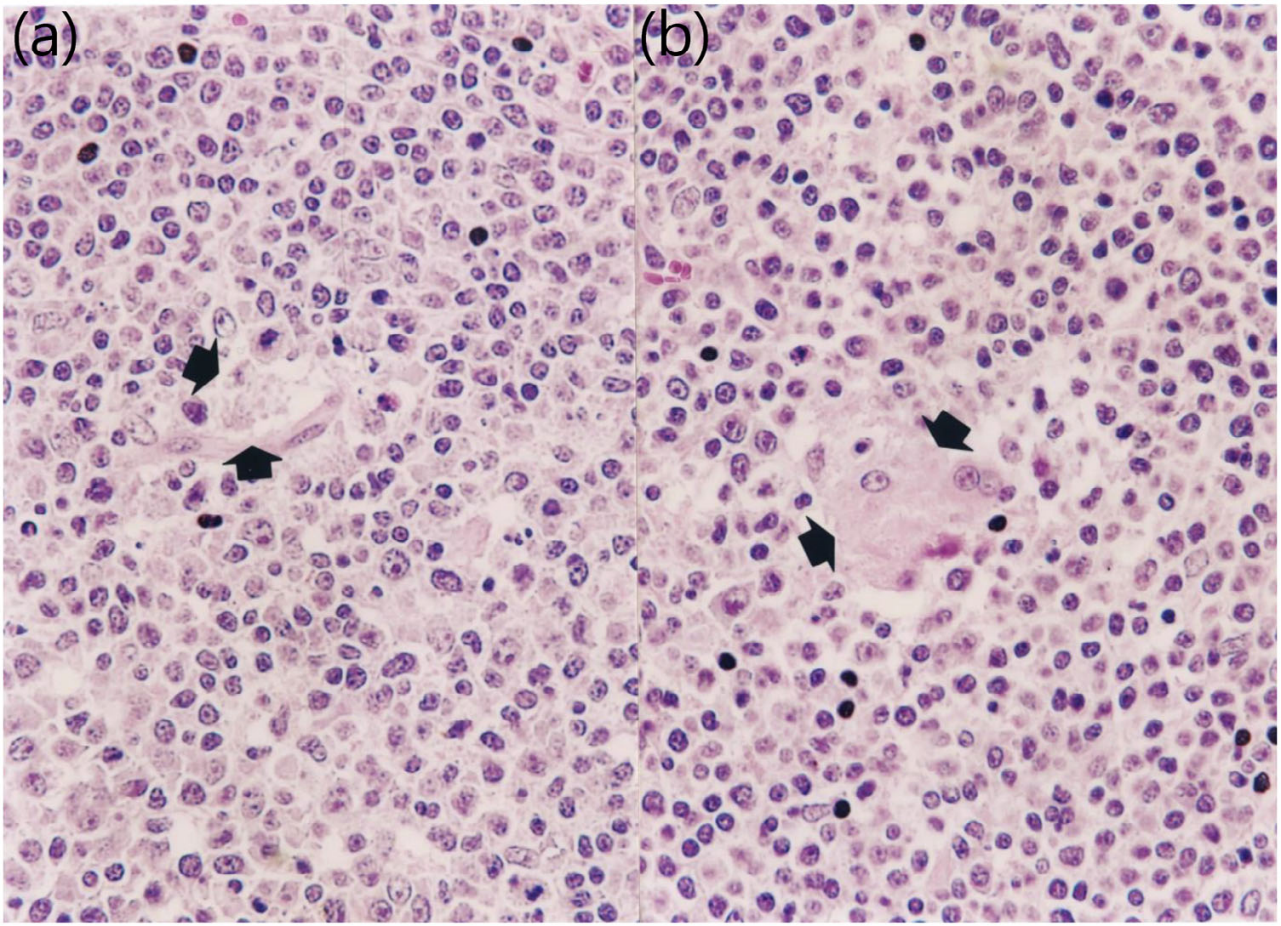
Histopathology of vaccinated pig (a) and unvaccinated pig (b) from farm A. Vaccinated pig had mild lymphoid depletion (arrows) and unvaccinated pig had moderate granulomatous inflammation (arrows).

In farms A, B and C, vaccination of animals significantly (*p* < 0.05) reduced the severity of macroscopic and microscopic lung lesions and microscopic lymphoid lesions (Figure 6a,b) compared to unvaccinated animals at 91 (112 days of age) and 154 (175 days of age) dpv. Vaccination of animals significantly (*p *< 0.05) reduced the number of PCV2 antigen‐positive cells in lymph nodes compared to unvaccinated animals at 91 (112 days of age), and 154 (175 days of age) dpv (Table 1).

## 4 DISCUSSION

The evaluated field trial conferred protection against subclinical PCV2d infection and enzootic pneumonia in pigs that received a PCV2d and *M. hyopneumoniae* bivalent vaccine. The most common clinical characteristic of both subclinical PCV2 infection and enzootic pneumonia is growth retardation of the pigs. As a result, growth performance was considered the critical index for successfully evaluating the efficacy of a bivalent vaccine under field conditions. Vaccination of pigs with the evaluated bivalent vaccine improved the growth performance in all three swine herds suffering from subclinical PCV2d infection and enzootic pneumonia under field conditions. Commercially, raised pigs are continuously exposed and re‐exposed to PCV2d field strains throughout their production process, which is why the positive effect of vaccination on growth performance demonstrated in this field trial is clinically significant.

The evaluated bivalent vaccine containing PCV2d and *M. hyopneumoniae* elicited immune responses against field strains of PCV2d and *M. hyopneumoniae*, even though the precise protective immunity is not well known yet. PCV2d protection is often measured by level of neutralizing antibodies and IFN‐γ‐SC, both of which are well‐known immune responses that are responsible for the clearance of PCV2 in the blood (Fort et al., 2012; Martelli et al., 2011; Meerts et al., 2005, 2006). Bivalent vaccination of pigs induced a high level of neutralizing anti‐PCV2d antibodies and PCV2d‐specific IFN‐γ‐SC which resulted in the reduction of both PCV2d blood viral load and lymphoid lesion severity. All of these, in turn, have had a positive effect on pig growth performance.

The bivalent PCV2d and *M. hyopneumoniae* vaccine evaluated in this study reduced PCV2d load in both the nasal and faecal samples under field conditions to similar levels of previous experimental studies (Patterson et al., 2011). PCV2 reduction of nasal and faecal shedding is a clinically meaningful comparison, as the nasal route may be more effective in transmission than through the faecal route (Patterson et al., 2011). Transmission through nasal and faecal secretions has been suggested as a potential mode of horizontal spreading (Patterson et al., 2011). Vaccination would be an effective disease control tool by lowering the risk of transmission to other pigs by decreasing the amount of PCV2 circulating within the herd.

Pig tonsil tissue samples that produced a mean concentration between 10^4^ and 10^6^ PCV2 genome copies/ng of total DNA were considered subclinically infected (Segalés Calsamiglia et al., 2005). The PCV2 mean concentrations from tonsillar tissue per mL ranged from 0.56 × 10^1^ to 5 × 10^1^ PCV2 genomic copies/ng of total DNA in vaccinated animals and from 2.7 × 10^4^ to 9.4 × 10^5^ PCV2 genomic copies/ng of total DNA in unvaccinated animals from all three farms at 112 days of age (91 days post‐vaccination) for the present study. This indicates that the bivalent vaccine evaluated in these field trials conferred protection against subclinical PCV2 infection.

Humoral immunity has not been associated with *M. hyopneumoniae* protection (Djordjevic et al., 1997), but cell‐mediated immunity has been previously correlated (Thacker et al., 2000). Bivalent vaccination induced high levels of *M. hyopneumoniae*‐specific IFN‐γ‐SC that resulted in the reduction of mycoplasmal laryngeal loads and reduced the severity of mycoplasmal lung lesions, all of which contributed to improve pig growth performance.

Mycoplasma quantification in the lungs of live, naturally infected pigs, is an appropriate evaluation of disease, as *M. hyopneumoniae* infects the lower respiratory tract, although quantitative analysis may be limited. Laryngeal swabs offer a more practical alternative for collecting samples for *M. hyopneumoniae* analysis under field conditions. Laryngeal swabs were successfully used as a reliable sampling method for the early detection of *M. hyopneumoniae*, followed by bronchoalveolar lavage fluid and nasal swabs in live experimentally infected pigs, particularly during the acute period of infection (Pieters et al., 2017).

Optimal vaccination timing is critical in efficiently controlling PCV2 infection, yet this is a difficult process. For example, vaccine failure may occur if piglets are vaccinated too early due to the prevalence of high maternal antibody. Maternally derived antibodies (MDA) may interfere with vaccine efficacy in 3‐week‐old pigs, which is the traditional timeframe for piglet vaccination. The present field trial demonstrated that the bivalent vaccine elicited systemic humoral and cellular immune responses to PCV2d and *M. hyopneumoniae* even in the presence of MDA. Therefore, an MDA effect on active immunization after vaccination at 3 weeks of age is less likely to hamper the efficacy of this bivalent vaccine.

This study was the first field trial that evaluated a PCV2d and *M. hyopneumoniae* bivalent vaccine in multiple herds already suffering from subclinical PCV2d infection and enzootic pneumonia. This study was necessary as most of the commercially bivalent vaccines of PCV2 and *M. hyopneumoniae* available today are based on PCV2a and/or PCV2b (Um et al., 2022; Yang et al., 2020, 2021) although PCV2d is the most prevalent PCV2 genotype currently circulating throughout Asia (Dinh et al., 2021; Park & Chae, 2021; Thangthamniyom et al., 2017; Tsai et al., 2019; Yang et al., 2018). It has been also reported that PCV2d is more virulent than PCV2a or PCV2b (Oh et al., 2021). The efficacy of PCV2 vaccination may depend on the PCV2 field viruses that are in circulation when pigs are naturally exposed to the disease (Takahagi et al., 2010). An additional comparative field trial that evaluated and determined that a trivalent vaccine containing PCV2a/b and *M. hyopneumoniae* was more effective in improving growth performance than a bivalent vaccine containing PCV2a and *M. hyopneumoniae* (Um et al., 2021) provides additional information in the broader PCV2 genomic picture. PCV2d‐caused PCVAD has been reported in PCV2a‐vaccinated herds (Opriessnig et al., 2013; Ramos et al., 2015; Seo et al., 2014). Despite these occasional instances of vaccine failure, several similar commercial bivalent vaccines made up of various PCV2 genotype antigens are used globally and have proven as efficacious in controlling co‐infections with PCV2d and *M. hyopneumoniae*. Additional direct comparison studies are needed to determine efficacy differences among these bivalent vaccines containing different PCV2 genotype antigens in farms suffering from co‐circulating PCV2d and *M. hyopneumoniae*.

## AUTHOR CONTRIBUTIONS

**Sehyeong Ham and Jeongmin Suh**: Conceptualization; data curation; investigation; methodology. **Chonghan Kim**: Methodology; resources; visualization; software. **Byoung‐Joo Seo**: Software; Formal analysis. **Gyeong‐Seo Park**: Formal analysis. **Chanhee Chae**: Conceptualization; project administration; supervision; writing – review and editing.

## CONFLICT OF INTEREST STATEMENT

The authors declare no conflicts of interest with respect to their authorship.

### ETHICS STATEMENT

The protocol for this field study was approved by the Seoul National University Institutional Animal Care and Use Committee (SNU‐201216‐5).

### PEER REVIEW

The peer review history for this article is available at https://publons.com/publon/10.1002/vms3.70001.

## Data Availability

The data that support the findings of this study are available from the corresponding author upon reasonable request.

## References

[vms370001-bib-0001] Alarcon, P. , Rushton, J. , & Wieland, B. (2013). Cost of post‐weaning multi‐systemic wasting syndrome and *Porcine circovirus* type‐2 subclinical infection in England—An economic disease model. Preventive Veterinary Medicine, 110(2), 88–102. 10.1016/j.prevetmed.2013.02.010 23490147 PMC3652492

[vms370001-bib-0002] Beaver, B. V. , Reed, W. , Leary, S. , McKiernan, B. , Bain, F. , Schultz, R. , Bennet, B. T. , Pascoe, P. , Shull, E. , Cork, L. C. , Francis‐Loyd, R. , Amass, K. D. , Johnson, R. , Schmidt, R. H. , Underwood, W. , Thornton, G. W. , & Kohn, B. (2001). 2000 Report of the AVMA panel on euthanasia. Journal of the American Veterinary Medical Association, 218, 669–696. 10.2460/javma.2001.218.669 11280396

[vms370001-bib-0003] Chae, C. (2012). Commercial *Porcine circovirus* type 2 vaccines: Efficacy and clinical application. The Veterinary Journal, 194(2), 151–157. 10.1016/j.tvjl.2012.06.031 22841450

[vms370001-bib-0004] Chae, C. (2016). Porcine respiratory disease complex: Interaction of vaccination and *Porcine circovirus* type 2, porcine reproductive and respiratory syndrome virus, and *Mycoplasma hyopneumoniae* . The Veterinary Journal, 212, 1–6. 10.1016/j.tvjl.2015.10.030 27256017

[vms370001-bib-0005] Dinh, P. X. , Nguyen, M. N. , Nguyen, H. T. , Tran, V. H. , Tran, Q. D. , Dang, K. H. , Le, H. T. , Nguyen, N. T. T. , Nguyen, T. T. , & Do, D. T. (2021). *Porcine circovirus* genotypes and their copathogens in pigs with respiratory disease in southern provinces of Vietnam. Archives of Virology, 166, 403–411. 10.1007/s00705-020-04878-y 33392818

[vms370001-bib-0006] Djordjevic, S. P. , Eamens, G. J. , Romalis, L. F. , Nicholls, P. J. , Taylor, V. , & Chin, J. (1997). Serum and mucosal antibody responses and protection in pigs vaccinated against *Mycoplasma hyopneumoniae* with vaccines containing a denatured membrane antigen pool and adjuvant. Australian Veterinary Journal, 75(7), 504–511. 10.1111/j.1751-0813.1997.tb14383 9258425

[vms370001-bib-0007] Dubosson, C. R. , Conzelmann, C. , Miserez, R. , Boerlin, P. , Frey, J. , Zimmermann, W. , Häni, H. , & Kuhnert, P. (2004). Development of two real‐time PCR assays for the detection of *Mycoplasma hyopneumoniae* in clinical samples. Veterinary Microbiology, 102, 55–65. 10.1016/j.vetmic.2004.05.007 15288927

[vms370001-bib-0046] Fort, M. , Fernandes, L. T. , Nofrarias, M. , Díaz, I. , Sibila, M. , Pujols, J. , Mateu, J. , & Segales, J. (2009). Development of cell‐mediated immunity to porcine circovirus type 2 (PCV2) in caesarean‐derived, colostrum‐deprived piglets. Veterinary immunology and immunopathology, 129(1‐2), 101–107. 19167096 10.1016/j.vetimm.2008.12.024 PMC7127047

[vms370001-bib-0008] Fort, M. , Sibila, M. , Nofrarias, M. , Perez‐Martin, E. , Olvera, A. , Mateu, E. , & Segalés, J. (2012). Evaluation of cell‐mediated immune responses against *Porcine circovirus* type 2 (PCV2) cap and rep proteins after vaccination with a commercial PCV2 sub‐unit vaccine. Veterinary Immunology and Immunopathology, 150, 128–132. 10.1016/j.vetimm.2012.09.001 23010221

[vms370001-bib-0009] Franzo, G. , Cortey, M. , Segalés, J. , Hughes, J. , & Drigo, M. (2016). Phylodynamic analysis of *Porcine circovirus* type 2 reveals global waves of emerging genotypes and the circulation of recombinant forms. Molecular Phylogenetics and Evolution, 100, 269–280. 10.1016/j.ympev.2016.04.028 27114187

[vms370001-bib-0010] Franzo, G. , & Segales, J. (2018). *Porcine circovirus 2* (PCV‐2) genotype update and proposal of a new genotyping methodology. PloS ONE, 13(12), e0208585. 10.1371/journal.pone.0208585 30521609 PMC6283538

[vms370001-bib-0011] Guo, L. , Fu, Y. , Wang, Y. , Lu, Y. , Wei, Y. , Tang, Q. , Fan, P. , Liu, J. , Zhang, F. , Huang, L. , Liu, D. , Li, S. , Wu, H. , & Liu, C. (2012). A *Porcine circovirus* type 2 (PCV2) mutant with 234 amino acids in capsid protein showed more virulence in vivo, compared with classical PCV2a /b strain, PloS ONE, 7(7), e41463. 10.1371/journal.pone.0041463 22829951 PMC3400667

[vms370001-bib-0012] Halbur, P. G. , Paul, P. S. , Frey, M. L. , Landgraf, J. , Eernisse, K. , Meng, X. J. , Lum, M. A. , Andrews, J. J. , & Rathje, J. A. (1995). Comparison of the pathogenicity of two US porcine reproductive and respiratory syndrome virus isolates with that of the Lelystad virus. Veterinary Pathology, 32(6), 648–660. 10.1177/0300985895032006 8592800

[vms370001-bib-0013] Ham, S. , Suh, J. , Oh, T. , Kim, C. , Seo, B.‐J. , & Chae, C. (2023). Efficacy of a novel bivalent vaccine containing *Porcine circovirus* type 2d and *Mycoplasma hyopneumoniae* against a dual PCV2d and *Mycoplasma hyopneumoniae* challenge. Frontiers in Veterinary Science, 10, 1176091. 10.3389/fvets.2023.1176091 37565086 PMC10410152

[vms370001-bib-0014] Jeong, J. , Park, C. , Choi, K. , & Chae, C. (2015). Comparison of three commercial one‐dose *Porcine circovirus* type 2 (PCV2) vaccines in a herd with concurrent circulation of PCV2b and mutant PCV2b. Veterinary Microbiology, 177, 43–52. 10.1016/j.vetmic.2015.02.027 25790733

[vms370001-bib-0015] Jeong, J. , Kang, I. , Kim, S. , Park, K. H. , Park, C. , & Chae, C. (2018). Comparison of 3 vaccination strategies against porcine reproductive and respiratory syndrome virus, *Mycoplasma hyopneumoniae*, and *Porcine circovirus* type 2 on 3 pathogen challenge model. Canadian Journal of Veterinary Research, 82, 39–47. 29382967 PMC5764041

[vms370001-bib-0016] Kim, J. , & Chae, C. (2004). Expression of monocyte chemoattractant protein‐1 and macrophage inflammatory protein‐1 in *Porcine circovirus* 2‐induced granulomatous inflammation. Journal of Comparative Pathology, 131, 121–126. 10.1016/j.jcpa.2004.02.001 15276851

[vms370001-bib-0017] Maes, D. , Verdonck, M. , Deluyker, H. , & de Kruif, A. (1996). Enzootic pneumonia in pigs. Veterinary Quarterly, 18(3), 104–109. 10.1080/01652176.1996.9694628 8903144

[vms370001-bib-0018] Martelli, P. , Ferrari, L. , Morganti, M. , Angelis, D. E. , Bonilauri, P. , Guazzetti, S. , Caleffi, A. , & Borghetti, P. (2011). One dose of a *Porcine circovirus* 2 subunit vaccine induces humoral and cell‐mediated immunity and protects against *Porcine circovirus*‐associated disease under field conditions. Veterinary Microbiology, 149, 339–351. 10.1016/j.vetmic.2010.12.008 21216540

[vms370001-bib-0019] Meerts, P. , Van‐Gucht, S. , Cox, E. , Vandebosch, A. , & Nauwynck, H. J. (2005). Correlation between type of adaptive immune response against *Porcine circovirus* type 2 and level of virus replication. Viral Immunology, 18(2), 333–341. 10.1089/vim.2005.18.333 16035945

[vms370001-bib-0020] Meerts, P. , Misinzo, G. , Lefebvre, D. , Nielsen, J. , Bøtner, A. , Kristensen, C. S. , & Nauwynck, H. J. (2006). Correlation between the presence of neutralizing antibodies against *Porcine circovirus* 2 (PCV2) and protection against replication of the virus and development of PCV2‐associated disease. BMC Veterinary Research, 2, 1–11. 10.1186/1746-6148-2-6 16445856 PMC1386657

[vms370001-bib-0021] Oh, T. , Suh, J. , Park, K. H. , Yang, S. , Cho, H. , & Chae, C. (2021). A comparison of pathogenicity and virulence of three *Porcine circovirus* type 2 (PCV2) genotypes (a, b, and d) in pigs singularly inoculated with PCV2 and dually inoculated with *Mycoplasma hyopneumoniae* and PCV2. Pathogens, 10(8), 979. 10.3390/pathogens10080979 34451444 PMC8400386

[vms370001-bib-0022] Opriessnig, T. , Thacker, E. L. , Yu, S. , Fenaux, M. , Meng, X.‐J. , & Halbur, P. G. (2004). Experimental reproduction of postweaning multisystemic wasting syndrome in pigs by dual infection with *Mycoplasma hyopneumoniae* and *Porcine circovirus* type 2. Veterinary Pathology, 41(6), 624–640. 10.1354/vp.41-6-624 15557072

[vms370001-bib-0023] Opriessnig, T. , Xiao, C.‐T. , Gerber, P. F. , & Halbur, P. G. (2013). Emergence of a novel mutant PCV2b variant associated with clinical PCVAD in two vaccinated pig farms in the U.S. concurrently infected with PPV2. Veterinary Microbiology, 163, 177–183. 10.1016/j.vetmic.2012.12.019 23305615

[vms370001-bib-0024] Park, K. H. , & Chae, C. (2021). The prevalence of *Porcine circovirus* type 2e (PCV2e) in Korean slaughter pig lymph nodes when compared with other PCV2 genotypes. Transboundary and Emerging Diseases, 68(6), 3043–3047. 10.1111/tbed.13975 33406315

[vms370001-bib-0025] Patterson, A. R. , Ramamoorthy, S. , Madson, D. M. , Meng, X. J. , Halbur, P. G. , & Opriessnig, T. (2011). Shedding and infection dynamics of *Porcine circovirus* type 2 (PCV2) after experimental infection. Veterinary Microbiology, 149, 91–98. 10.1016/j.vetmic.2010.10.020 21111547

[vms370001-bib-0026] Pieters, M. , Daniels, J. , & Rovira, A. (2017). Comparison of sample types and diagnostic methods for in vivo detection of *Mycoplasma hyopneumoniae* during early stages of infection. Veterinary Microbiology, 203, 103–109. 10.1016/j.vetmic.2017.02.014 28619131

[vms370001-bib-0027] Pogranichnyy, R. M. , Yoon, K. J. , Harms, P. A. , Swenson, S. L. , Zimmerman, J. J. , & Sorden, S. D. (2000). Characterization of immune response of young pigs to *Porcine circovirus* type 2 infection. Viral Immunology, 13(2), 143–153. 10.1089/vim.2000.13.143 10892995

[vms370001-bib-0028] Ramos, N. , Mirazo, S. , Castro, G. , & Arbiza, J. (2015). First identification of porcine pircovirus type 2b mutant in pigs from Uruguay. Infection, Genetics and Evolution, 33, 320–323. 10.1016/j.meegid.2015.05.02 26004195

[vms370001-bib-0029] Segalés, J. , Allan, D. , & Domingo, M. (2005). *Porcine circovirus* diseases. Animal Health Research Reviews, 6, 119–142. 10.1079/ahr2005106 16583778

[vms370001-bib-0030] Segalés, J. , Calsamiglia, M. , Olvera, A. , Sibila, M. , Badiella, L. , & Domingo, M. (2005). Quantification of *Porcine circovirus* type 2 (PCV2) DNA in serum and tonsillar, nasal, trachea‐bronchial, urinary and faecal swabs of pigs with and without postweaning multisystemic wasting syndrome (PMWS). Veterinary Microbiology, 111, 223–229. 10.1016/j.vetmic.2005.10.008 16289542

[vms370001-bib-0031] Segalés, J. (2012). *Porcine circovirus* type 2 (PCV2) infections: Clinical signs, pathology and laboratory diagnosis. Virus Research, 164, 10–19. 10.1016/j.virusres.2011.10.007 22056845

[vms370001-bib-0032] Seo, H. W. , Park, C. , Kang, I. , Choi, K. , Jeong, J. , Park, S. J. , & Chae, C. (2014). Genetic and antigenic characterization of a newly emerging *Porcine circovirus* type 2b mutant first isolated in cases of vaccine failure in Korea. Archives of Virology, 159, 3107–3111. 10.1007/s00705-014-2164-6 25034669

[vms370001-bib-0033] Shen, H. G. , Beach, N. M. , Huang, Y. W. , Halbur, P. G. , Meng, X. J. , & Opriessnig, T. (2010). Comparison of commercial and experimental *Porcine circovirus* type 2 (PCV2) vaccines using a triple challenge with PCV2, porcine reproductive and respiratory syndrome virus (PRRSV), and porcine parvovirus (PPV). Vaccine, 28(37), 5960–5966. 10.1016/j.vaccine.2010.07.002 20637768

[vms370001-bib-0034] Takahagi, Y. , Toki, S. , Nishiyama, Y. , Morimatsu, F. , & Murakami, H. (2010). Differential effects of *Porcine circovirus* type 2 (PCV2) vaccination on PCV2 genotypes at Japanese pig farms. Journal of Veterinary Medical Science, 72(1), 35–41. 10.1292/jvms.09-0314 19915335

[vms370001-bib-0035] Thacker, E. L. , Thacker, B. J. , Kuhn, M. , Hawkins, P. A. , & Waters, W. R. (2000). Evaluation of local and systemic immune responses induced by intramuscular injection of a *Mycoplasma hyopneumoniae* bacterin to pigs. American Journal of Veterinary Research, 61(11), 1384–1389. 10.2460/ajvr.2000.61.1384 11108184

[vms370001-bib-0036] Thangthamniyom, N. , Sangthong, P. , Poolperm, P. , Thanantong, N. , Boonsoongnern, A. , Hansoongnern, P. , Semkum, P. , Petcharat, N. , & Lekcharoensuk, P. (2017). Genetic diversity of *Porcine circovirus* type 2 (PCV2) in Thailand during 2009–2015. Veterinary Microbiology, 208, 239–246. 10.1016/j.vetmic.2017.08.006 28888644

[vms370001-bib-0045] Tsai, G. T. , Lin, Y. C. , Lin, W. H. , Lin, J. H. , Chiou, M. T. , Liu, H. F. , & Lin, C. N. (2019). Phylogeographic and genetic characterization of porcine circovirus type 2 in Taiwan from 2001–2017. Scientific Reports, 9(1), 10782. 31346205 10.1038/s41598-019-47209-1 PMC6658515

[vms370001-bib-0038] Um, H. , Yang, S. , Oh, T. , Park, K. H. , Cho, H. , Suh, J. , Min, K.‐D. , & Chae, C. (2021). Comparative evaluation of growth performance between bivalent and trivalent vaccines containing *Porcine circovirus* type 2 (PCV2) and *Mycoplasma hyopneumoniae* in a herd with subclinical PCV2d infection and enzootic pneumonia. Vaccines, 9, 450. 10.3390/vaccines9050450 34063582 PMC8147604

[vms370001-bib-0039] Um, H. , Yang, S. , Oh, T. , Cho, H. , Park, K. H. , Suh, J. , & Chae, C. (2022). A field efficacy trial of trivalent vaccine containing *Porcine circovirus* type 2a and 2b, and *Mycoplasma hyopneumoniae* in three herds. Veterinary Medicine and Science, 8(2), 578–590. 10.1002/vms3.657 34687172 PMC8959324

[vms370001-bib-0040] Xiao, C. T. , Halbur, P. G. , & Opriessnig, T. (2012). Complete genome sequence of a novel *porcine circovirus* type 2b variant present in cases of vaccine failures in the United States. Journal of Virology, 86(22), 12469. 10.1128/jvi.02345-12 23087125 PMC3486456

[vms370001-bib-0041] Xiao, C. T. , Halbur, P. G. , & Opriessnig, T. (2015). Global molecular genetic analysis of *porcine circovirus* type 2 (PCV2) sequences confirms the presence of four main PCV2 genotypes and reveals a rapid increase of PCV2d. Journal of General Virology, 96(7), 1830–1841. 10.1099/vir.0.000100 25711965

[vms370001-bib-0042] Yang, S. , Yin, S. , Shang, Y. , Liu, B. , Yuan, L. , Zafar Khan, M. U. , Liu, X. , & Cai, J. (2018). Phylogenetic and genetic variation analyses of *porcine circovirus* type 2 isolated from China. Transboundary and Emerging Diseases, 65, e383–e392. 10.1111/tbed.12768 29150903

[vms370001-bib-0043] Yang, S. , Oh, T. , Park, K. H. , Cho, H. , & Chae, C. (2020). A dual swine challenge with *Porcine circovirus* type 2 (PCV2) and *Mycoplasma hyopneumoniae* used to compare a combination of mixable monovalent PCV2 and monovalent *M. hyopneumoniae* vaccines with a ready‐to use PCV2 and *M. hyopneumoniae* bivalent vaccine. Frontiers in Veterinary Science, 7, 579. 10.3389/fvets.2020.00579 32984414 PMC7492382

[vms370001-bib-0044] Yang, S. , Ahn, Y. , Oh, T. , Cho, H. , Park, K. H. , & Chae, C. (2021). Field evaluation of a single‐dose bivalent vaccine of *Porcine circovirus* type 2b and *Mycoplasma hyopneumoniae* . Veterinary Medicine and Science, 7, 755–765. 10.1002/vms3.420 33386664 PMC8136929

